# New implants in orthopaedics – *caveat emptor*

**DOI:** 10.1308/003588412X13373405386853

**Published:** 2012-10

**Authors:** D Warwick, IA Trail

**Affiliations:** ^1^University Hospital Southampton NHS Foundation Trust,UK; ^2^Wrightington, Wigan and Leigh NHS Foundation Trust,UK

**Keywords:** Joint replacement, Arthroplasty, Hand, Wrist

Joint replacement in orthopaedic surgery has developed immeasurably over the past four decades, enhancing the lives of millions each year across the world. Well established hip and knee replacements offer 20-year survivorship or more with few post-operative complications.

Nevertheless, there are examples of joint implantation causing unexpected harm. Some metal-on-metal hip replacements are now a great cause for concern with systemic metal ion accumulation and irretrievable loosening. Certain small joint replacements in the hand have proven to be unreliable with high complication rates and worrying early failure rates.^1,2^ Another paper reporting an implant for the wrist appears in this issue of the *Annals*.^3^ This has stimulated us to present our views here: we believe that despite laudable intentions to help a patient with apparently cutting edge surgery, some surgeons may be enticed by clever marketing or personal ambition into using implants with insufficient evidence of safety.

What is insufficient evidence? This is not just absence of evidence but includes: small case series; follow-up too short to detect even early failure; publication from an enthusiastic proponent, perhaps involved in the design of the implant; insensitive or irrelevant outcome measures; and single centre series in which the enthusiasm, expertise and learning curve may not be generalisable to the average surgeon. It is striking how the literature seems to contain numerous short-term follow-up studies for certain devices yet no subsequent long-term reviews. Might this be explained by the enthusiasm of both authors and journals to publish something new and exciting?

It is, of course, incumbent on us as clinicians to advance our orthopaedic knowledge but this has to be within a framework of proper audit, governance and research. As new implants emerge, they must be subject to strict evaluation. It is mandatory for the surgeon to receive critical peer review of the potential advantage of this new implant over established treatment. This may mean ethics committee guidance together with institutional governance approval. After all, the institution will be liable for poor outcomes on procedures performed on their premises.

The outcomes must be then measured systematically using a combination of subjective patient related outcomes and objective radiological assessment. This assessment must continue indefinitely to establish survivorship. However, on many occasions an individual surgeon is unlikely to accumulate enough experience to detect poor outcomes or limited survivorship. The National Joint Registry has led the way with hip and knee replacements, generating invaluable data on implants, centres and surgeons. This registry should be expanded to cover all implants.

Finally, the use of implants with a very rare indication or complex technical requirements should be concentrated in just a few specialised centres. Otherwise dilution means that no surgeon gets past his or her learning curve and, inevitably, more patients are exposed to potential harm. Concentration of resource needs the support of the professional associations and healthcare commissioners to overcome inevitable tensions.
